# In Vitro and In Vivo Inhibition of Intestinal Glucose Transport by Guava (*Psidium Guajava*) Extracts

**DOI:** 10.1002/mnfr.201701012

**Published:** 2018-05-17

**Authors:** Ulrike Müller, Flora Stübl, Bettina Schwarzinger, Georg Sandner, Marcus Iken, Markus Himmelsbach, Clemens Schwarzinger, Nicole Ollinger, Verena Stadlbauer, Otmar Höglinger, Tobias Kühne, Peter Lanzerstorfer, Julian Weghuber

**Affiliations:** ^1^ University of Applied Sciences Upper Austria 4600 Wels Austria; ^2^ Austrian Competence Center for Feed and Food Quality Safety and Innovation 4600 Wels Austria; ^3^ PM International AG 5445 Schengen Luxembourg; ^4^ Johannes Kepler University Institute for Analytical Chemistry 4040 Linz Austria; ^5^ Johannes Kepler University Institute for Chemical Technology of Organic Materials 4040 Linz Austria

**Keywords:** GLUT2, guava extract, intestinal glucose transport, postprandial blood glucose, SGLT1

## Abstract

**Scope:**

Known pharmacological activities of guava (*Psidium guajava*) include modulation of blood glucose levels. However, mechanistic details remain unclear in many cases.

**Methods and results:**

This study investigated the effects of different guava leaf and fruit extracts on intestinal glucose transport in vitro and on postprandial glucose levels in vivo. Substantial dose‐ and time‐dependent glucose transport inhibition (up to 80%) was observed for both guava fruit and leaf extracts, at conceivable physiological concentrations in Caco‐2 cells. Using sodium‐containing (both glucose transporters, sodium‐dependent glucose transporter 1 [SGLT1] and glucose transporter 2 [GLUT2], are active) and sodium‐free (only GLUT2 is active) conditions, we show that inhibition of GLUT2 was greater than that of SGLT1. Inhibitory properties of guava extracts also remained stable after digestive juice treatment, indicating a good chemical stability of the active substances. Furthermore, we could unequivocally show that guava extracts significantly reduced blood glucose levels (≈fourfold reduction) in a time‐dependent manner in vivo (C57BL/6N mice). Extracts were characterized with respect to their main putative bioactive compounds (polyphenols) using HPLC and LC‐MS.

**Conclusion:**

The data demonstrated that guava leaf and fruit extracts can potentially contribute to the regulation of blood glucose levels.

## 1 Introduction

Diabetes mellitus is a chronic disease that ultimately leads to hyperglycemia. In this context, people with type 2 diabetes mellitus (T2DM) constitute the majority of people (>90%) with diabetes around the world.^1^ T2DM is a global public health crisis threatening the economies of all nations. According to WHO, the global prevalence of diabetes among adults over 18 years of age has risen from 4.7% (108 million people) in 1980 to 8.5% (422 million people) in 2014.

Hyperglycemia, insulin resistance, and obesity are typical features of T2DM. T2DM has been frequently reported to also be associated with hyperlipidemia and hypertension. The combination of these circumstances is also known as metabolic syndrome, which is a high risk factor for cardiovascular diseases.^2^, ^3^ Therefore, developing new strategies for treatment and prevention of these health problems is of great importance. One possible strategy is the direct manipulation of blood glucose levels via insulin mimetic substances and enhanced glucose transporter 4 (GLUT4) activities.^4^, ^5^ We have recently developed an in ovo model to test the efficacy of such compounds also in a living organism.^6^, ^7^ Other options are based on strategies to lower nutrient absorption. Specifically, inhibited or delayed intestinal glucose absorption may have a tremendous impact on managing diabetes and obesity.

Intestinal glucose absorption is predominantly facilitated by sodium‐dependent glucose transporter 1 (SGLT1),^8^, ^9^ whereas glucose efflux from enterocytes into the blood is mediated by glucose transporter 2 (GLUT2).^10^ Nevertheless, it has been recently shown that GLUT2 can be recruited into the apical membrane under high luminal glucose concentrations, leading to bulk absorption of glucose by facilitated diffusion.^11^, ^12^, ^13^, ^14^ Therefore, GLUT2‐mediated glucose absorption represents a high‐potential target for effective regulation of the blood glucose level.

Convincing evidence suggests that selected phytochemicals can directly affect intestinal glucose uptake by competitive inhibition of certain sugar transporters. Bioactive compounds such as polyphenols, phenolic acids, and tannins are of particular importance in this context and have been reported to interact with SGLT1 and GLUT2.^15^, ^16^, ^17^, ^18^, ^19^, ^20^, ^21^

Guava (*Psidium guajava*) is an important food crop and medicinal plant in tropical and subtropical countries. Relevant phytochemicals include phenols, polyphenols, and carotenoids, as well as triterpenes and terpenoids. Several pharmacological studies report on antioxidative, antiallergic, antimicrobial, and antidiabetic effects of guava leaf and fruit extracts.^22^ However, knowledge of the pharmacological mechanism of action is limited. Furthermore, the effects of guava extracts on blood glucose levels are controversial. Some in vitro and in vivo studies have indicated hypoglycemic effects of guava leaves and their extracts via enhanced insulin secretion^23^, ^24^ and elevated hepatic glucose uptake,^25^ respectively. The antihyperglycemic properties of guava leaf extract (GLE) are also supported by two previous studies.^26^, ^27^ In contrast, Sungawa and colleagues did not support this evidence and even found a blood glucose‐increasing effect of guava leaves in a long‐term study in rats.^28^ Furthermore, there is little understanding of guava fruit extracts in this context.^29^

Here, we tested the effects of different guava extracts (commercially available as well as homemade leaf and fruit extracts) on intestinal glucose transport in vitro and in vivo. In human intestinal Caco‐2 cells, our data indicate that guava extracts at physiological concentrations are potential inhibitors of SGLT1 and GLUT2. The inhibitory effect was also confirmed after digestive juice treatment, which implies chemical stability of bioactive components. Moreover, antihyperglycemic properties of guava extracts, via reducing the postprandial glucose response, were proved in vivo (C57BL/6N mice). Taken together, these results indicate that consumption of guava extracts may contribute to moderate regulation of blood glucose levels by direct inhibition of intestinal glucose transport.

## 2 Experimental Section

### 2.1 Reagents

MEM with Earle's salts, DMEM, fetal bovine serum (FBS), trypsin‐EDTA, and antibiotics were purchased from Biochrom GmbH (Berlin, Germany). Entero‐STIM Intestinal Epithelium Differentiation Medium and MITO+ Serum Extender were purchased from Corning (Wiesbaden, Germany). Hesperetin, peltatoside, hyperoside, kaempferol, ellagic acid, isoquercitrin, quercitrin, phloretin, (−)‐epicatechin, epigallocatechin, epicatechin gallate, procyanidin B1, procyanidin B2, epigallocatechin gallate, gallocatechin, and morine were obtained from Extrasynthese (Genay Cedex, France). Phloridzin, chlorogenic acid, caffeic acid, quercetin, (+)‐catechin, gallic acid, 2‐mercaptoethanol, NaCl, MgSO_4_, KCl, CaCl_2_, HEPES, and xylitol were purchased from Sigma‐Aldrich (Schnelldorf, Germany). Guaijaverin and avicularin were obtained from Glentham Life Sciences (Corsham, United Kingdom). Purified and concentrated GLE (cGLE) was provided by Belan GmbH (Wels, Austria).^30^ Guava fruit puree for preparation of fruit extracts was obtained from Tradework BV (Rotterdam, The Netherlands), commercially available GLE and apple extract (AE) were purchased from Pfannenschmidt (Hamburg, Germany). Transwell inserts (12 mm, collagen‐treated, 0.4 μm pore size) and 12‐well plates were obtained from Corning (Wiesbaden, Germany).

### 2.2 Cell Culture and Differentiation

Human Caco‐2 cells were purchased from DSMZ (Braunschweig, Germany). Cells were maintained in MEM with Earle's salts supplemented with 10% FBS, 100 μg mL^−1^ penicillin/streptomycin, and 0.1% 2‐mercaptoethanol and grown at 37 °C in a humidified atmosphere (≥95%) with 5% CO_2_. For transport studies, cells were seeded at 5.6 × 10^5^ cells/insert in Entero‐STIM Intestinal Epithelium Differentiation Medium supplemented with 1% penicillin/streptomycin and 0.1% MITO+ Serum Extender. Cell medium was changed daily, and the experiment was carried out between day 5 and 7. Differentiation of the monolayer was assessed by measuring transepithelial electrical resistance (TEER) of the cell monolayers. Only transwell inserts with a resistance exceeding a blank membrane by 300 Ω were utilized in the experiments.

### 2.3 Preparation of Guava Fruit Extracts

#### 2.3.1 Ethanolic Extraction

Ethanolic guava fruit extract (GFE_EtOH_) was prepared by drying guava fruit puree at 60 °C for 72 h and crushing it into powder with a grinder. For ethanolic extraction, 0.5 g of the guava fruit powder was stirred into 25 mL of EtOH (80%) for 10 min at 60 °C. Mixtures were filtered, and the procedure was repeated with powder residues. Ethanolic extracts were then concentrated by rotary evaporation and collected in 3 mL of deionized water.

#### 2.3.2 Supercritical Fluid Extraction

Supercritical fluid extraction was performed on guava puree after drying in a vacuum chamber at 40 °C to remove residual water content of 10%. Extraction was performed with carbon dioxide at a temperature of 40 °C under a pressure of 30 MPa, and precipitation of the extract was conducted at 25 °C and 4 MPa. The material was extracted for 3 h in a continuous mode using fresh CO_2_ and venting the exhaust after precipitation of the extract (supercritical guava fruit extract [GFE_SFE_]).

### 2.4 Cytotoxicity Assay

Cytotoxic effects of compounds under study were evaluated by using a resazurin‐based in vitro toxicology assay (Sigma‐Aldrich; Schnelldorf, Germany), according to the instructions of the manufacturer. Briefly, cells were seeded into 96‐well plates (30 000 cells per well), grown to 90% confluence, and incubated with the test substances for 4 h at 37 °C. Subsequently, the cells were washed and incubated with a medium containing 10% resazurin for 2 h. Levels of the reduced form of resazurin (resorufin) were then determined using a microplate reader in fluorescence mode (544 nm excitation, 590 nm emission; POLARstar Omega, BMG LABTECH, Ortenberg, Germany). Data were analyzed using OmegaMARS Data analysis software package (BMG LABTECH, Ortenberg, Germany). Cell viability was normalized to untreated cells grown under the same conditions. Each test substance was measured in triplicate.

### 2.5 Glucose Transport Assay

For experiments, medium was discarded, and differentiated cells were washed twice with HEPES buffer (20 mM HEPES, 137 mM NaCl, 4.7 mM KCl, 1.2 mM MgSO_4_, 1.8 mM CaCl_2_) and placed into a new 12‐well plate containing 800 μL of HEPES buffer in the basolateral compartment. The apical compartment was then filled with 500 μL of donor solution consisting of cell culture medium with 2.1 g L^−1^ glucose, 1.0 g L^−1^ xylitol and the substance of interest at indicated concentrations. Next, 100 μL of samples were collected from the basolateral compartment at various time points and analyzed for their glucose content using HPLC analysis. TEER values were examined at each time point to ensure cell monolayer integrity. Finally, 100 μL of donor solution from the apical compartment were used to quantitate the remaining glucose concentration.

For the treatment of guava extracts with digestive juices, 100 mg of extract was incubated with 1 mL of gastric juice (NaCl 2.9 g L^−1^, KCl 0.7 g L^−1^, KH_2_PO_4_ 0.27 g L^−1^, pepsin 1 g L^−1^, and mucin 3 g L^−1^; pH 2) or chyle (KCl 0.3 g L^−1^, CaCl_2_ 0.5 g L^−1^, MgCl_2_ 0.2 g L^−1^, NaHCO_3_ 1 g L^−1^, trypsin 0.3 g L^−1^, pancreatin 9 g L^−1^, bile 9 g L^−1^, and urea 0.3 g L^−1^; pH 7.5) for 2 h at 37 °C; then, samples were diluted to a final concentration of 100 mg L^−1^ in culture medium with 2.1 g L^−1^ glucose and 1.0 g L^−1^ xylitol, and the transport study was carried out as described.

Except for dose–response study, a concentration of 100 mg L^−1^ for GLEs (solid) and 100 mL L^−1^ for guava fruit extracts (liquid) were used in the experiments. Therefore, extract doses were at a comparable m/v concentration with regard to phloretin/phloridzin control experiments (100 mg L^−1^).

### 2.6 Determination of Glucose and Xylitol Content by HPLC

Sugar analysis was carried out as previously reported with minor modifications.^31^ A Jasco LC‐2000 Plus Series system comprised of an analytical pump with external degasser, autosampler, temperature‐controlled column compartment, a Jasco RI‐2031 Plus detector and a UV‐Vis detector equipped with Chrompass software (all from Jasco Corporation, Tokyo, Japan) was used. Analysis of glucose and xylitol was conducted using the same HPLC system. Separation was performed on a Varian, Meta Carb 87H (PN A5210, SN 12509907) column. The column temperature was set to 56 °C, and isocratic elution was carried out at 0.8 mL min^−1^. A mobile phase of 5 mM sulfuric acid in ddH_2_O was used. HPLC was calibrated with glucose (ranging from 10 to 1000 mg L^−1^) and xylitol (5 to 1000 mg L^−1^). The obtained standard curves were linear within this range for both, glucose and xylitol. The limit of detection (LOD) was defined as a signal‐to‐noise ratio of 2:1 and limit of quantitation (LOQ) as 4:1. LOD was 2.5 mg L^−1^ and LOQ 5 mg L^−1^ for glucose and xylitol, respectively. Data were processed by Jasco Chrompass Chromatography System software (version 1.7.403.1).

### 2.7 Identification and Quantitation of Polyphenols by HPLC

Identification and quantitation of polyphenols was conducted as previously reported with minor modifications.^32^ HPLC analysis was performed on an Agilent 1260 Infinity LC System equipped with vacuum degasser, quaternary pump, autosampler, and photodiode array detector. High‐resolution mass spectra were obtained using a Thermo Fisher Scientific LTQ Orbitrap XL with an Ion Max API Electrospray Source operated in negative ionization mode. The following parameters were used in this analysis: capillary temperature, 350 °C; sheath gas flow, 45 U; aux gas flow, 15 U; source voltage, 3.5 kV; capillary voltage, −25 V; tube lens, −90 V. Separations were carried out using a Hypersil ODS C18 column (250 mm × 4.6 mm inner diameter, 5 μm particle size; Thermo Fisher Scientific, Vienna, Austria). Analytes were separated by gradient elution with (A) 0.1% formic acid (FA) in water and (B) acetonitrile containing 0.1% formic acid. The injection volume was 5 μL for all samples, and the flow rate was set to 0.67 mL min^−1^ with a gradient adapted to constant elution.

Identified polyphenols in cGLE, GLE, GFE_EtOH_, and AE were quantitated by reversed‐phase chromatography using a LaChrom Elite HPLC System (VWR‐Hitachi) comprised of a L‐2100 pump with built‐in degasser, a L‐2200 cooled autosampler, a temperature‐controlled column compartment, and a L‐2455 diode array detector (DAD) equipped with an EZ Chrom Elite Chromatography data system. Analyte separation was performed on a Hypersil ODS C18 column (250 mm × 4.6 mm inner diameter, 5 μm particle size; Thermo Fisher Scientific, Vienna, Austria). The column temperature was set to 30 °C, and the injection volume was 20 μL for all samples. UV wavelengths were detected at 280 nm and 320 nm. Analytes were separated by gradient elution with mobile phase A containing 0.1% trifluoroacetic acid (TFA) in water and mobile phase B containing 0.1% TFA in acetonitrile at a flow rate of 1 mL min^−1^. Elution gradient starting conditions were 95% A and 5% B. The proportion of B was increased to 30% at 40 min and to 50% at 50 min, followed by 80% B for 5 min from 50.1 to 55 min and 5% B for 5 min from 55.1 to 60 min. Polyphenols were quantified against known standards and were available with concentrations in a linear range of 1 to 1000 mg L^−1^.

Analysis of GFE_SFE_ was carried out by reversed‐phase chromatography using a Thermo Scientific Dionex Ultimate 3000 comprised of a LPG‐3400SD pump with built‐in degasser, a WPS‐3000 U(T)SL cooled autosampler, a temperature‐controlled column compartment, and an FLD‐34000RS diode array detector (DAD) equipped with Chromeleon software. Analyte separation was performed on an Accucore C18 column (150 mm × 3.0 mm inner diameter, 2.6 μm particle size; Thermo Scientific). The column temperature was set to 40 °C, and the injection volume was 1 μL. UV wavelengths were detected at 260 nm and 360 nm. Analytes were separated by gradient elution with mobile phase A containing 0.1% FA in water and mobile phase B containing 0.1% FA in acetonitrile at a flow rate of 0.5 mL min^−1^. Elution gradient starting conditions were 95% A and 5% B. After 5 min equilibration time, the proportion of B was increased to 20% at 8 min and to 40% at 12 min, followed by 60% B at 15 min and 80%B at 17 min for 3 min. B was reduced to 5% for 20–25 min.

### 2.8 qPCR

Expression values of GLUT2, glucose transporter 5 (GLUT5), SGLT1, claudin‐1 (CLDN1), claudin‐3 (CLDN3), and occludin (OCLN) were analyzed in the Caco‐2 model system on differentiation days 2–7 using qPCR (C1000 Thermal Cylcer and CFX96 Real‐Time System, BioRad). Total RNA was isolated in triplicates per day (QIAShredder and RNeasy Mini Kit, Qiagen), followed by reverse‐transcription of 50 ng total RNA (end volume: 20 μL) into cDNA (iScript cDNA Synthesis Kit, BioRad) and qPCR (iQ SYBR Green Supermix, BioRad). For qPCR, 10 μL iQ SYBR Green Supermix (2×), 2 μL primer mix forward/reverse (each 3 pmol μL^−1^), 6 μL nuclease‐free water and 2 μL of a cDNA pool sample (triplicates per day) were used. The qPCR protocol included 3 min at 95 °C for initial activation followed by 40 PCR cycles using a denaturation for 15 s at 95 °C and an annealing step for 15 s at 62 °C. After each cycle, a plate reading step was executed. Finally, a melt curve analysis was carried out by gradually increasing the temperature for 0.5 °C starting from 65 to 95 °C. The determined *C*_t_ values were normalized to the housekeeping gene expression of HPRT1 and calculated to relative mRNA expression levels using the 2^−∆∆C^_T_ method.^33^ A list of the used primers is shown in Table S1, Supporting Information. The qPCR products were also analyzed via agarose gel electrophoresis. For this purpose, the qPCR products were premixed with 2 μL Midori Green Direct (Nippon Genetics) and run on a 2% agarose gel at 110 V for 30 min.

### 2.9 Animal Model and Treatment

Female C57BL/6N mice were received from Janvier Labs at the age of 8 weeks and conventionally housed at 22 °C and a 12‐h light:12‐h dark cycle with free access to water and food (Rat/Mouse Maintenance Chow, 10 mm, V1534‐000, Ssniff GmbH, Germany). Animals were initially divided into three groups consisting of six mice each. To allow for acclimation to the local environment, mice were kept in the animal room for at least 24 days without treatment, except for general husbandry. The day before oral glucose tolerance tests (OGTTs) were performed, mice were transferred to the procedure room, and were withdrawn from food for 12 h, except for water. Test solutions were prepared freshly on each test day. OGTT was performed on two days, with 6 mice on each test day divided into the following groups: (A) control (1.5 g glucose per kg bodyweight); (B) GLE (1.5 g glucose + 400 mg cGLE per kg bodyweight) and (C) GFE (1.5 g glucose + 400 mg GFE_SFE_ per kg bodyweight). Extract doses were chosen based on several studies investigating the effect of phytogenic substances on post‐prandial glucose response.^34^, ^35^, ^36^, ^37^, ^38^ Test solutions were orally administered by gavage, and blood samples were collected from the tail tip before and 30, 60, 90, and 120 min after glucose load with subsequent blood glucose measurements using a glucometer Contour XT instrument (Bayer, Germany). All experiments were approved by the Austrian Animal Ethics Committee (reference number: BMWFW‐66.012/0018‐WF/V/3b/2017).

### 2.10 Calculations and Statistics

The area under the curve (AUC) of glucose in mice experiments was calculated for each experimental condition as the area beneath the curve and above the fasting level from 0 to 120 min using GraphPad Prism 6.0 for Windows (GraphPad Software, Inc., San Diego, CA, USA). Significance testing was performed using GraphPad Prism 6.0 for Windows. Differences were considered significant with *p* < 0.05 for two‐way ANOVA followed by Tukey's multiple comparisons test. All values are presented as the means ± SE, if not otherwise stated.

## 3 Results

### 3.1 Setup of an Accelerated 7‐Day Caco‐2 Glucose Transport Model

The human epithelial colorectal adenocarcinoma cell‐line Caco‐2 has been reported to express high levels of the relevant intestinal glucose transporters SGLT1 and GLUT2.^39^ Therefore, we used these cells to set up a stable in vitro intestinal transport model based on a transwell system. Due to inconsistent literature concerning culturing and experimental conditions for accelerated Caco‐2 differentiation, we characterized suitable control parameters to ensure the reliability of the assay.

**Figure 1:**
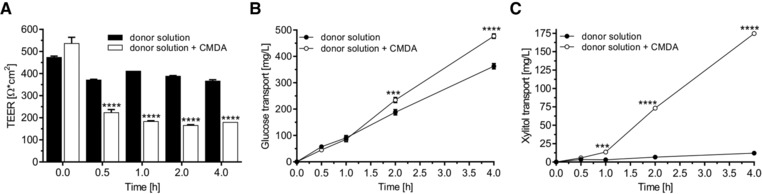
Effects of the cell monolayer‐disturbing agent (CMDA) tomatine (5 mg L^−1^) on defined control parameters. Caco‐2 cells were grown on collagen‐coated 0.4 μm transwell inserts for monolayer formation and fast differentiation. On days 5–7, glucose transport across the cell monolayer was quantitated. Cell culture medium with 2.1 g L^−1^ glucose and 1.0 g L^−1^ xylitol was placed as donor solution in the apical compartment. Samples were collected from the basolateral compartment (HEPES buffer) at the respective time points. Glucose and xylitol concentrations of the samples were measured by HPLC. A) Influence of CMDA on the membrane integrity as evaluated by TEER measurements. B) Effect of CMDA on the cumulative glucose and C) xylitol transport from the apical to the basolateral side of Caco‐2 monolayers. Error bars are based on the standard error of the mean (*n* = 8 inserts, measured on four different days) *p < 0.05, **p < 0.01, ***p < 0.001, ****p < 0.0001.

First, differentiation state was investigated by gene expression analysis of the important tight junction proteins CLDN1 and CLDN3 as well as OCLN (Figure S2D–F, Supporting Information). Furthermore, expression of the sugar transporters GLUT2, SGLT1, as well as GLUT5 was investigated (Figure S2A–C, Supporting Information). The chosen number of cells that were seeded led to the formation of a confluent cell monolayer already on differentiation day 2. Our data clearly show relevant expression of selected glucose transporters and tight junction proteins on differentiation days 5–7. Second, TEER measurements were carried out before each experiment and at defined time points during the transport study to determine the influence of the substances under study on the monolayer integrity of differentiated Caco‐2 cells. The cell monolayer‐disturbing agent (CMDA) tomatine (5 mg L^−1^) was used as a control substance (**Figure** 1). We could show that the cytotoxic effect of this compound leads to an immediate significant decrease of the TEER (Figure 1A) compared to untreated cells (readily detectable after 0.5 h, *p* < 0.0001), thus confirming defective membrane integrity. Third, quantitation of cumulative glucose transport by HPLC further confirmed these disruptive mechanisms: Tomatine treatment led to a significant increase in glucose transport (≈480 mg L^−1^ after 4 h, *p* < 0.0001) across the cell monolayer compared to untreated cells (≈350 mg L^−1^ after 4 h) (Figure 1B). Fourth, the sugar alcohol xylitol, which has been reported to be poorly absorbed in the small intestine,^40^, ^41^ was used as another control substance. As shown in Figure 1C, only minor amounts of xylitol were found in the basolateral chamber of untreated cells. By contrast, tomatine treatment led to an immediate massive increase of xylitol (significantly elevated xylitol values detectable after 1 h [*p* < 0.001], with steady increases up to 4 h [*p* < 0.0001]). Based on these results, appropriately stable TEER values (more than 300 Ω) and low cumulative xylitol diffusion (glucose:xylitol ratio >10:1) over the observation period were used as quantitative control parameters for each experiment. Finally, phloretin and phloridzin were used as reference substances for inhibiting intestinal glucose transport across the Caco‐2 monolayer. These polyphenolic compounds are known to be effective SGLT1 and GLUT2 inhibitors.^42^, ^43^ Both substances (100 mg L^−1^) caused a decrease in glucose transport compared to untreated cells, whereas phloretin (≈80%) led to a more prominent inhibition than phloridzin (≈20%) (Figure S1A, Supporting Information). Calculated values for apparent permeability of these conditions are shown in Figure S1B, Supporting Information. Non‐toxic conditions for all substances used in this study were approved by cytotoxicity tests (data not shown).

### 3.2 Concentrated Guava Leaf Extract Inhibits Glucose Transport under Sodium‐Dependent Conditions in Caco‐2 Cells

**Figure 2:**
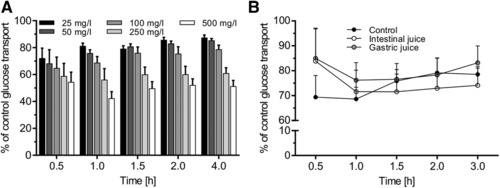
Effects of cGLE on intestinal glucose transport inhibition under sodium‐dependent conditions. Caco‐2 cells were grown on collagen‐coated 0.4 μm transwell inserts for monolayer formation and fast differentiation. On days 5–7, glucose transport across the cell monolayer was quantitated. Cell culture medium with 2.1 g L^−1^ glucose and 1.0 g L^−1^ xylitol was placed as donor solution in the apical compartment. Samples were collected from the basolateral compartment (HEPES buffer) at the respective time points. Glucose concentration of the samples was measured by HPLC. A) Dose‐dependent inhibition of intestinal glucose transport across the Caco‐2 monolayer by cGLE at indicated concentrations. B) Determination of the remaining effect of cGLE on intestinal glucose transport after intestinal or gastric juice incubation. A total of 100 mg of cGLE was dissolved in 1 mL of intestinal or gastric juice for 2 h at 37 °C and diluted to a final concentration of 250 mg L^−1^ in culture medium with 2.1 g L^−1^ glucose and 1.0 g L^−1^ xylitol, and the transport study was carried out as described. Error bars are based on the standard error of the mean (*n* = 10 inserts, measured on 5 different days).

GLEs have been reported to exhibit hypoglycemic properties via enhancing insulin secretion and elevating hepatic glucose uptake. Here, we further report on a putative mechanism via inhibition of intestinal glucose transport using a flavonol‐enriched GLE as flavonols have been previously reported to inhibit GLUT2‐mediated intestinal glucose transport.^18^ In the presence of Na^+^ (main intestinal glucose transporters SGLT1 and GLUT2 are active), the cumulative glucose transport across the Caco‐2 monolayer from the apical compartment (2.1 g L^−1^ glucose in donor solution) to the basolateral compartment within 4 h was found to be ≈350 mg L^−1^ buffer solution (Figure 1B). Addition of cGLE (purified for flavonols using preparative chromatography,^30^ at varying concentrations ranging from 25 to 500 mg L^−1^, to the apical side led to a dose‐dependent inhibition of the basolateral transport of glucose (**Figure** 2A). The maximal inhibitory effect was reached in cells treated with a concentration of 500 mg L^−1^ of cGLE in the apical chamber. Interestingly, glucose transport was also inhibited in a time‐dependent manner and did not appear to be constant over time, as the maximal inhibitory effect (≈60%) occurred 1 h after cGLE addition and decreased to ≈50% after 4 h.

A prerequisite for effective inhibition of intestinal glucose transport is the stability of the respective substance against degradation processes occurring in the digestive tract. Otherwise, a loss of bioactive function is very likely. For this purpose, we simulated digestive processes by incubating the cGLE (100 mg L^−1^) with intestinal or gastric juice for 2 h, followed by glucose transport experiments. Digestive juice treatment of cGLE resulted in a slightly reduced glucose transport inhibition compared to experiments with undigested cGLE. However, digested extracts still exhibited a prominent inhibitory activity compared to untreated cells (Figure 2B).

### 3.3 Inhibition of Glucose Transport by cGLE under Sodium‐Free Conditions in Caco‐2 Cells

**Figure 3:**
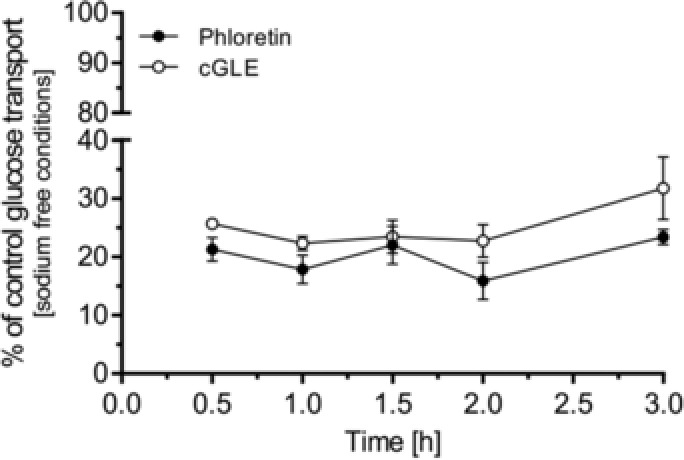
Inhibitory effect of cGLE on glucose transport under sodium‐free conditions. Caco‐2 cells were grown on collagen‐coated 0.4 μm transwell inserts for monolayer formation and fast differentiation. On days 5–7, glucose transport across the cell monolayer was quantitated. HEPES buffer (sodium chloride was replaced with potassium chloride) with 2.1 g L^−1^ glucose and 1.0 g L^−1^ xylitol as well as the indicated extract (100 mg L^−1^) was used as a donor solution in the apical compartment. Samples were collected from the basolateral compartment (HEPES buffer) at the respective time points. Glucose concentration of the samples was measured by HPLC. Error bars are based on the standard error of the mean (*n* = 10 inserts, measured on 5 different days).

The same experiments as described in Section 3.2 were repeated under sodium‐free conditions. Sodium chloride in HEPES buffer was replaced by potassium chloride. We expected SGLT1 inactivity and exclusive GLUT2‐mediated apical and basolateral glucose transport under these conditions.^19^ Compared to sodium‐dependent conditions (Figure 2B; 100 mg L^−1^ cGLE, ≈31% inhibition after 30 min and ≈22% after 3 h), cGLE showed a significantly higher inhibitory effect (100 mg L^−1^ cGLE, ≈74% after 30 min and 68% after 3 h), similar to the known GLUT2 inhibitor phloretin (100 mg L^−1^ phloretin, ≈79% inhibition after 30 min and 77% after 3 h) (**Figure** 3). These results indicate that GLUT2 is the primary apical surface target of cGLE, and the absence of sodium does not greatly affect the pattern of inhibition.

### 3.4 Guava Leaf and Fruit Extracts Inhibit Intestinal Glucose Transport in Caco‐2 Cells

**Figure 4:**
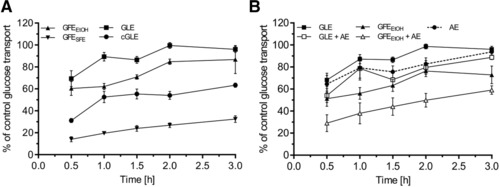
Efficacy of different guava extracts on inhibition of glucose transport and additive effect of AE. Caco‐2 cells were grown on collagen‐coated 0.4 μm transwell inserts for monolayer formation and fast differentiation. On days 5–7, glucose transport across the cell monolayer was quantitated. Cell culture medium with 2.1 g L^−1^ glucose and 1.0 g L^−1^ xylitol as well as the indicated extract was placed as a donor solution in the apical compartment. Samples were collected from the basolateral compartment (HEPES buffer) at the respective time points. Glucose concentration of the samples was measured by HPLC. A) Comparison of glucose transport‐inhibiting properties of different guava extracts. A total of 100 mg L^−1^ indicated guava leaf extracts (GLE and cGLE) and 100 mL L^−1^ guava fruit extract (GFE_EtOH_ and GFE_SFE_) were tested. B) Synergistic effects on glucose transport inhibition by combined treatment with AE (100 mg L^−1^). Error bars are based on the standard error of the mean (*n* = 10, measured on 5 different days).

Leaf and fruit extracts have been reported to affect blood glucose levels^24^, ^26^, ^27^, ^29^; however, detailed mechanisms remain to be elucidated. Based on the identified inhibitory effects of cGLE on glucose transport in Caco‐2 cells, we next investigated the influence of a GLE and two differently prepared fruit extracts (GFE_EtOH_ and GFE_SFE_). Especially guava fruit extracts are of pivotal interest as there is hardly any relevant information available. GFE_EtOH_ is an ethanolic extract that was concentrated by rotary evaporation, whereas GFE_SFE_ was concentrated via supercritical fluid extraction. Compared to cGLE, both GLE (100 mg L^−1^) and GFE_EtOH_ (100 mL L^−1^) showed reduced but significant inhibitory properties that occurred in a time‐dependent manner with an extent of ≈30–40% (30 min) to ≈10% (3 h) (**Figure** 4A). Interestingly, GFE_SFE_ (100 mL L^−1^) exhibited the most prominent inhibitory property with a maximum glucose transport inhibition of ≈87% (30 min). This even exceeded the inhibitory effect of the well‐known SGLT1 and GLUT2 inhibitor phloretin (Figure S1, Supporting Information).

### 3.5 Additive Inhibitory Effect of Guava Extract and AE

Polyphenol‐rich AE has been reported to effectively inhibit intestinal glucose transport^19^ and to reduce postprandial blood glucose levels.^21^ Here, we further investigated possible additive effects of a commercially available AE with the minor powerful inhibitory effect of GLE and GFE_EtOH_ on glucose transport to determine whether extract combinations can enhance inhibitory properties. For this purpose, cells were treated with guava extract and AE in various combinations (Figure 4B). AE alone showed a similar inhibitory potential as GLE (≈35% inhibition after 30 min and 10% after 3 h), but in combination with GLE and GFE_EtOH_, glucose transport inhibition could be further increased by ≈20% when compared to conditions with single extracts.

### 3.6 Guava Extracts Diminish the Postprandial Glucose Response in C57BL/6N Mice

To prove that the putative antidiabetic properties of guava leaf and fruit extracts in a physiological context, we next investigated the influence of guava extract administration on the postprandial glucose response in mice. For these experiments, cGLE and GFE_SFE_ were chosen because these extracts seemed to be the most promising candidates with regard to glucose transport inhibition in the Caco‐2 cell model. To assess the effects of the 12‐h starvation period on the mouse physiology, body weight and blood glucose levels were determined before and after the fasting period. The body weight of all animals remained stable (Figure S3A, Supporting Information), whereas blood glucose levels slightly decreased over the fasting period (Figure S3B, Supporting Information). Nevertheless, both parameters were at a comparable level for the cohort of mice examined, and therefore, the 12‐h fasting period was considered acceptable in terms of both physiology and animal welfare.

**Figure 5:**
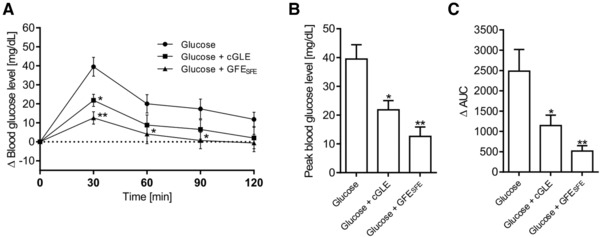
Effect of a single dose of guava leaf and fruit extract co‐administered with glucose on postprandial glucose response in female C57BL/6N mice. A) Changes (Δ) in mean incremental concentrations of blood glucose. B) Mean peak blood glucose levels. C) Calculated area under the curve (Δ). Data are shown as the mean of *n* = 6 mice ± SE. *p < 0.05, **p < 0.01, ***p < 0.001.

Significant differences between the control and experimental groups were found in the postprandial glucose response (Δ) at various time points (**Figure** 5A). Plasma glucose levels reached peak values 30 min after oral glucose gavage in the control group and both experimental groups. Thereafter, glucose concentrations gradually declined over the remaining test period. Significantly decreased peak blood glucose levels (Δ) were detected for both extracts (cGLE: 21.8 ± 3.2 mg dL^−1^, *p* < 0.05; GFE_SFE_:12.6 ± 3.3 mg dL^−1^, *p* < 0.01), when compared to the control group (39.5 ± 4.9 mg dL^−1^) (Figure 5B). Based on the postprandial glucose response, mean areas under the curve (ΔAUCs) were calculated for all three experimental groups. Again, cGLE and GFE_SFE_ resulted in a significantly decreased ΔAUC when compared to the glucose control group (Figure 5C).

### 3.7 Identification and Quantitation of Polyphenols in Guava Extracts and AE

**Figure 6:**
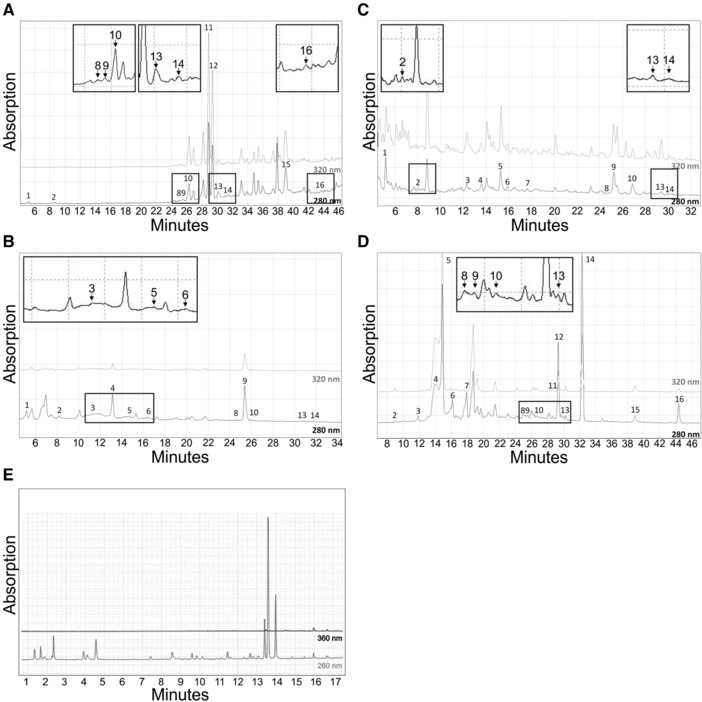
HPLC‐DAD chromatogram of A) cGLE, B) GLE, C) GFE_EtOH_, D) AE, and E) GFE_SFE_ recorded at indicated wavelengths. For peak numbers, refer to Table 1.

Based on the reported glucose transport inhibitory properties of polyphenols,^16^, ^17^, ^18^, ^19^, ^20^ in this study, we analyzed the polyphenolic content of guava extract and AE using HPLC. First, compounds were identified using mass spectrometry and UV spectra followed by quantitation using calibration curves of relevant standards. Except for GFE_SFE_, up to 16 polyphenolic compounds were identified and quantified depending on extract purity and concentration in the guava leaves and fruit extracts: phloridzin and phloretin (dihydrochalcone derivates), quercetin, quercitrin, isoquercitrin, hyperoside, avicularin and guaijaverin (flavonols), procyanidin B1 and B2, (+)‐catechin, (−)‐epicatechin, gallocatechin, epicatechin gallate (flavan‐3‐ols), and gallic and ellagic acid (hydroxybenzoic acids). Isoquercitrin and hyperoside values are presented as the sum of both. Representative HPLC‐DAD diagrams, indicating retention times and maximal wavelengths of each compound, are shown in **Figure** 6 for (A) cGLE, (B) GLE, (C) GFE_EtOH_, and (E) GFE_CO2_. **Table** 1 summarizes the content of identified polyphenolics in respective guava extracts. cGLE is especially rich in flavonols (≈2609 mg L^−1^), with guaijaverin (728 mg L^−1^) and quercetin (698 mg L^−1^) as the main substances. All other polyphenolic groups were only found at very low concentrations. In GLE and GFE_EtOH_, mainly flavan‐3‐ols (≈850 and ≈595 mg L^−1^, respectively) and hydroxybenzoic acids (≈280 mg L^−1^ and ≈174 mg L^−1^) were found. Surprisingly, in GFE_SFE_, none of the polyphenols identified in the other guava extracts could be verified or assigned.

**Table 1:** Identification of phenolic compounds in guava and apple extracts (AE) using HPLC with DAD and Orbitrap MS

Peak number	Retention time, *t*_R_ [min]	Compound	Mass spectrometry [m/z]	cGLE [mg L^−1^]	GLE [mg L^−1^]	GFE_EtOH_ [mg L^−1^]	GFE_SFE_ [mg L^−1^]	AE [mg L^−1^]
Hydroxycinnamic acids								
5	14.6	Chlorogenic acid	354.0957	n.d.	47	14	n.d.	1420
Dihydrochalcone derivates								
14	31.8	Phloridzin	436.1376	35	n.d.	1.2	n.d.	719
16	43.5	Phloretin	274.0847	45	n.d.	n.d.	n.d.	47
Flavan‐3‐ols								
3	12.0	Procyanidin B1	578.1430	n.d.	310	124	n.d.	252
6	16.5	Procyanidin B2	578.1430	n.d.	81	118	n.d.	312
4	13.5	(+)‐Catechin	290.0797	n.d.	236	78	n.d.	1765
7	17.6	(–)‐Epicatechin	290.0797	n.d.	n.d.	46	n.d.	527
2	8.2	Gallocatechin	306.0746	100	215	214	n.d.	79
8	24.9	Epicatechingallate	442.0906	16	7.2	11	n.d.	97
Flavonols								
13	30.1	Quercitrin	448.1012	162	2.3	20	n.d.	46
10	26.2	Isoquercitrin/hyperoside	464.0961	451	7.0	50	n.d.	129
11	28.8	Guaijaverin	434.0855	728	n.d.	n.d.	n.d.	29
12	29.3	Avicularin	434.0855	586	n.d.	n.d.	n.d.	205
15	38.5	Quercetin	302.0433	698	n.d.	n.d.	n.d.	112
Hydrobenzoic acids								
9	25.3	Ellagic acid	302.0069	27	258	113	n.d.	100
1	5.2	Gallic acid	170.1110	2.4	21	60.9	n.d.	n.d.
		Total		2856	1188	855	—	5847

In AE, 16 polyphenols, belonging to five different major polyphenolic groups: chlorogenic acid (hydroxycinnamic acids), phloridzin and phloretin (dihydrochalcone derivates), procyanidin B1 and B2, (+)‐catechin, (−)‐epicatechin, epicatechin gallate, and gallocatechin (flavan‐3‐ols) and quercetin, quercitrin, isoquercitrin, hyperoside, and avicularin (flavonols), were identified and quantified. In addition, the hydroxybenzoic acid ellagic acid was also identified. Isoquercitrin and hyperoside are presented as the sum of both. A representative HPLC‐DAD diagram, indicating retention times and maximal wavelengths of each compound, is shown in Figure 6D. Table 1 summarizes the content of identified polyphenolics in AE. Among the five investigated polyphenolic groups, the flavan‐3‐ols group was found to be the most abundant (≈2780 mg L^−1^), with (+)‐catechin being the main compound in this group (1765 mg L^−1^). Hydroxycinnamic acids represent the second largest group of polyphenols detected in the investigated AE (≈1425 mg L^−1^). Chlorogenic acid was the most abundant polyphenolic substance in this group, with a mean concentration of 1420 mg L^−1^. Furthermore, two dihydrochalcone derivates were identified: phloridzin (719 mg L^−1^) and phloretin (48 mg L^−1^). Ellagic acid (100 mg L^−1^, hydroxybenzoic acid) and substances of the flavonol group (≈490 mg L^−1^) were only detected at lower concentrations compared to other constituents.

Single polyphenols identified in cGLE, GLE, and AE are consistent with previous reports,^32^, ^44^, ^45^ whereas the concentration and the total amount quantitated by HPLC shows large variations due to different extract preparation and further purification steps. To our knowledge, a detailed single polyphenol analysis for guava fruit extracts has not yet been published.

## 4 Discussion

Caco‐2 monolayers are commonly used in basic research and in the pharmaceutical industry as an in vitro model of the human small intestinal mucosa to predict the absorption of administered drugs.^46^, ^47^, ^48^ Here, we used this model to study the influence of guava extracts on intestinal glucose transport across the Caco‐2 monolayer. A prerequisite for the simulation of in vivo intestinal processes are differentiated cell monolayers expressing tissue‐typical cell membrane and transport proteins.^49^ Several strategies have been reported that mainly vary in differentiation time, ranging from 3 days^50^, ^51^, ^52^ to up to 3 weeks,^50^, ^53^ and media supplements. Due to this inhomogeneity of differentiation protocols, we set up a well‐characterized Caco‐2 monolayer model based on fast differentiated cells (within 5–7 days) to minimize cell culture periods and maximize experimental throughput. Based on gene expression analysis of various sugar transporters (GLUT2, GLUT5, and SGLT1) as well as tight junction proteins (CLDN1, CLDN3, and OCLN), we could clearly show that a sufficient amount of relevant proteins are expressed upon shortened differentiation time (Figure S2, Supporting Information). We are aware that based on relative expression, no direct correlation between total amounts of mRNA can be drawn. However, distinct conclusions can be obtained by comparing the *C*_t_ values obtained from qPCR (Table S2, Supporting Information), which indicate stable and comparable target expression already from differentiation days 2–7. Furthermore, based on literature, we defined a TEER value exceeding a blank transwell membrane by 300 Ω as the minimum prerequisite for all experiments. Furthermore, we set the maximum threshold ratio (glucose:xylitol) for cumulative xylitol diffusion to be 10:1 within 4 h, as xylitol has been reported not be absorbed in the lumen in large amounts.^40^, ^41^ In contrast, cumulative glucose transport within 4 h experiment time was defined to be between 300 and 400 mg L^−1^ buffer solution. In addition, we used the known SGLT1 and GLUT2 inhibitors phloretin and phloridzin^42^, ^43^ to further prove the use of fast‐differentiated Caco‐2 cells for studying intestinal glucose transport.

The influence of dietary polyphenols on sugar absorption and digestion has been extensively studied.^54^ Several reports have indicated that single polyphenols inhibit glucose and fructose transport by interacting with sugar transporters in various ways.^18^, ^55^, ^56^, ^57^, ^58^ Furthermore, several polyphenol‐rich plant‐ and fruit‐derived extracts have been reported to effectively inhibit intestinal glucose uptake and transport.^15^, ^17^, ^19^, ^59^, ^60^, ^61^, ^62^, ^63^, ^64^ In this context, as GLEs are rich in polyphenols,^30^, ^44^, ^65^ they were shown to have antihyperglycemic properties by acting via various mechanisms.^24^, ^25^, ^26^, ^27^ However, inhibition of intestinal glucose transport by GLEs has not yet been investigated. Furthermore, the role of guava fruit extracts is currently unknown in this regard. We, therefore, investigated the intestinal glucose transport inhibition properties of different guava leaf and fruit extracts in the Caco‐2 cell model. Here, we provide the first evidence that both guava leaf and fruit extracts are able to reduce intestinal glucose transport. As GLUT2 and SGLT1 are the main transporters for glucose in the small intestine,^54^ reduced glucose flux is most likely caused by direct inhibition of those transporters in Caco‐2 cells. This assumption is in line with a previous study, where polyphenol‐rich fruit extracts were shown to inhibit GLUT2 and SGLT1.^19^ Nevertheless, a possible influence on other transporters cannot be completely excluded, and the subcellular site of inhibition still needs to be determined. However, variation of sodium concentration in transport experiments suggests that the inhibition of GLUT2, which is the major glucose transporter under sodium‐free conditions and high luminal glucose concentrations,^12^ by cGLE is greater than inhibition of SGLT1 (Figure 3). cGLE is especially rich in flavonols, which were previously reported to specifically inhibit glucose transport via GLUT2 in *Xenopus laevis* oocytes.^18^ Glucose transport inhibition could be further increased by combinatorial application of guava leaf/fruit and polyphenol‐rich AE (Figure 5). Our data indicate that flavonols have more prominent glucose transport inhibition properties than flavan‐3‐ols, as the flavonol‐rich cGLE is more effective than the flavan‐3‐ol‐rich GFE_EtOH_, GLE, and even AE at comparable experimental extract concentrations. By combining flavan‐3‐ol‐rich guava extract and AE, a similar additive inhibitory effect could be achieved. However, further experiments are necessary to unravel the detailed molecular inhibition mechanisms as well as to identify the individual bioactive polyphenolic fractions. Digestive juice treatment only led to a slightly reduced inhibitory effect when compared to untreated GLE (Figure 2). This indicates that putative bioactive compounds are resistant against enzymatic degradation or that metabolites exhibit similar inhibitory properties.

In addition to the in vitro transwell approach, we further investigated the influence of guava fruit and leaf extracts on the postprandial glucose response in vivo. Here, we could unequivocally show that cGLE and GFE_SFE_ significantly reduce blood glucose levels in a time‐dependent manner when compared to the glucose control group in C57BL/6N mice. These are the first results reporting on a possible novel strategy for blood glucose control by guava extracts via inhibiting intestinal glucose transport.

Phytochemical analysis of the different guava extracts demonstrated large variations in single polyphenol composition. In general, the identified polyphenols are consistent with previous reports: Well‐known main phenolic constituents of *P. guajava* leaves include quercetin, quercitrin, isoquercitrin, guaijaverin, avicularin, hyperoside, ellagic acid, procyanidin A and B, catechin, gallocatechin, gallic acid, delphinidin, myricetin, morine, guavinoside, guavin, and naringenin.^30^, ^44^, ^65^ Most of these compounds were also identified in guava leaf and fruit extracts in this study (Table 1). Surprisingly, we were not able to identify any polyphenolic compounds by mass spectrometry in GFE_SFE_. Polyphenolic supercritical fluid extraction has been so far only reported for guava leaf^66^ and guava seeds,^67^ but to our knowledge, no data are available for guava fruits. As the GFE_SFE_ exhibited the most prominent inhibitory property when compared to the other extracts in this study, we speculate that there might be other bioactive guava fruit constituents and polyphenols that still need to be identified and investigated.

Results from recent studies reveal evidence that GLUT2 has a fundamental role in intestinal glucose transport across the brush border membrane of enterocytes.^11^, ^12^, ^13^, ^14^ Our data indicate the potential of guava leaf and fruit extracts to inhibit GLUT2 and SGLT1 and therefore reduce intestinal glucose transport in vitro and in vivo. Important strategies for the prevention and regulation of hyperglycemia include the exploration for insulin mimetic substances and direct manipulation of the major blood glucose regulator GLUT4,^4^, ^5^ as well as the exploration for compounds that reduce glucose uptake in the small intestine by inhibiting transporters such as the SGLT1 and GLUT2.^17^, ^18^, ^19^, ^20^ Our results indicate that consumption of not only guava leaf but also fruit extracts and functionalized beverages might be a potential source for dietary polyphenols and other bioactive compounds that are able to effectively inhibit glucose transport in the small intestine.

## Conflict of Interest

The authors declare no conflict of interest. PM International AG provided support in the form of salaries for authors M.I. and T.K. but did not have any additional role in the study design.

## Supporting information

Supporting Information

Supporting Information Figure S1

Supporting Information Figure S2

Supporting Information Figure S3

## References

[1] S. Smyth , A. Heron , Nature Med. 2006, 12, 75. 16397575 10.1038/nm0106-75

[2] P. Dandona , A. Aljada , A. Chaudhuri , P. Mohanty , R. Garg , Circulation 2005, 111, 1448. 15781756 10.1161/01.CIR.0000158483.13093.9D

[3] R. H. Eckel , S. M. Grundy , P. Z. Zimmet , Lancet 2005, 365, 1415. 15836891 10.1016/S0140-6736(05)66378-7

[4] P. Lanzerstorfer , V. Stadlbauer , L. A. Chtcheglova , R. Haselgrübler , D. Borgmann , J. Wruss , P. Hinterdorfer , K. Schroder , S. M. Winkler , O. Hoglinger , J. Weghuber , Br. J. Pharmacol. 2014, 171, 5237. 25039620 10.1111/bph.12845 PMC4262000

[5] V. Stadlbauer , R. Haselgrübler , P. Lanzerstorfer , B. Plochberger , D. Borgmann , J. Jacak , S. M. Winkler , K. Schroder , O. Hoglinger , J. Weghuber , PloS One 2016, 11, e0148109. 26820984 10.1371/journal.pone.0148109 PMC4731058

[6] R. Haselgrübler , F. Stübl , K. Essl , M. Iken , K. Schroder , J. Weghuber , PloS One 2017, 12, e0182788. 28777818 10.1371/journal.pone.0182788 PMC5544204

[7] R. Haselgrübler , F. Stübl , V. Stadlbauer , P. Lanzerstorfer , J. Weghuber , J. Vis. Exp. 2018, https://doi.org/10.3791/57237. 10.3791/57237 PMC6100700 29733303

[8] N. Harada , N. Inagaki , J. Diabetes Investig 2012, 3, 352. 10.1111/j.2040-1124.2012.00227.x PMC4019254 24843589

[9] E. M. Wright , D. D. Loo , B. A. Hirayama , Physiol. Rev. 2011, 91, 733. 21527736 10.1152/physrev.00055.2009

[10] M. Mueckler , Eur. J. Biochem. 1994, 219, 713. 8112322 10.1111/j.1432-1033.1994.tb18550.x

[11] A. Au , A. Gupta , P. Schembri , C. I. Cheeseman , Biochem. J. 2002, 367, 247. 12095416 10.1042/BJ20020393 PMC1222871

[12] G. L. Kellett , E. Brot‐Laroche , Diabetes 2005, 54, 3056. 16186415 10.2337/diabetes.54.10.3056

[13] W. R. McClure , T. M. Jovin , J. Biol. Chem. 1975, 250, 4073. 1092683

[14] E. L. Morgan , O. J. Mace , J. Affleck , G. L. Kellett , J. Physiol. 2007, 580, 593. 17272350 10.1113/jphysiol.2006.124768 PMC2075547

[15] F. Alzaid , H. M. Cheung , V. R. Preedy , P. A. Sharp , PloS One 2013, 8, e78932. 24236070 10.1371/journal.pone.0078932 PMC3827299

[16] C. H. Chen , H. J. Hsu , Y. J. Huang , C. J. Lin , Planta Med. 2007, 73, 348. 17511059 10.1055/s-2007-967172

[17] T. L. Farrell , S. L. Ellam , T. Forrelli , G. Williamson , BioFactors 2013, 39, 448. 23361943 10.1002/biof.1090

[18] O. Kwon , P. Eck , S. Chen , C. P. Corpe , J. H. Lee , M. Kruhlak , M. Levine , FASEB J. 2007, 21, 366. 17172639 10.1096/fj.06-6620com

[19] S. Manzano , G. Williamson , Mol. Nutr. Food Res. 2010, 54, 1773. 20564476 10.1002/mnfr.201000019

[20] M. Pinent , M. Blay , M. C. Blade , M. J. Salvado , L. Arola , A. Ardevol , Endocrinology 2004, 145, 4985. 15271880 10.1210/en.2004-0764

[21] C. Schulze , A. Bangert , G. Kottra , K. E. Geillinger , B. Schwanck , H. Vollert , W. Blaschek , H. Daniel , Mol. Nutr. Food Res. 2014, 58, 1795. 25074384 10.1002/mnfr.201400016

[22] R. M. Gutierrez , S. Mitchell , R. V. Solis , J. Ethnopharmacol. 2008, 117, 1. 18353572 10.1016/j.jep.2008.01.025

[23] A. Hassan , M. Bahrani , H. Zaheri , N. Soltani , F. Kharazmi , M. Keshavarz , M. Kamalinajad , J. Diabetes Mellit. 2012, 2, 138.

[24] D. K. Obatomi , E. O. Bikomo , V. J. Temple , J. Ethnopharmacol. 1994, 43, 13. 7967645 10.1016/0378-8741(94)90111-2

[25] F. C. Cheng , S. C. Shen , J. S. Wu , J. Food Sci. 2009, 74, H132. 19646046 10.1111/j.1750-3841.2009.01149.x

[26] J. A. Ojewole , Methods Find. Exp. Clin. Pharmacol. 2005, 27, 689. 16395418 10.1358/mf.2005.27.10.948917

[27] S. C. Shen , F. C. Cheng , N. J. Wu , Phytother. Res. 2008, 22, 1458. 18819164 10.1002/ptr.2476

[28] M. Sunagawa , S. Shimada , Z. Zhang , A. Oonishi , M. Nakamura , T. Kosugi , J. Health Sci. 2004, 50, 674.

[29] S. Kumari , R. Rakavi , M. Mangaraj , J. Clin. Diagn. Res. 2016, 10, BC04. 10.7860/JCDR/2016/21291.8425 PMC5071920 27790420

[30] T. Eidenberger , M. Selg , K. Krennhuber , Fitoterapia 2013, 89, 74. 23707747 10.1016/j.fitote.2013.05.015

[31] J. Wruss , G. Waldenberger , S. Huemer , P. Uygun , P. Lanzerstorfer , U. Müller , O. Höglinger , J. Weghuber , J. Food Composit. Anal. 2015, 42, 46.

[32] P. Lanzerstorfer , J. Wruss , S. Huemer , A. Steininger , U. Muller , M. Himmelsbach , D. Borgmann , S. Winkler , O. Hoglinger , J. Weghuber , J. Agric. Food Chem. 2014, 62, 1047. 24410208 10.1021/jf4051232

[33] K. J. Livak , T. D. Schmittgen , Methods 2001, 25, 402. 11846609 10.1006/meth.2001.1262

[34] L. A. F. Afiune , T. Leal‐Silva , Y. K. Sinzato , R. Q. Moraes‐Souza , T. S. Soares , K. E. Campos , R. T. Fujiwara , E. Herrera , D. C. Damasceno , G. T. Volpato , PloS One 2017, 12, e0179785. 28644857 10.1371/journal.pone.0179785 PMC5482446

[35] C. V. Rynjah , N. N. Devi , N. Khongthaw , D. Syiem , S. Majaw , J. Tradit. Complement. Med. 2018, 8, 134. 29322001 10.1016/j.jtcme.2017.04.007 PMC5755985

[36] T. B. Tafesse , A. Hymete , Y. Mekonnen , M. Tadesse , BMC Complement. Altern. Med. 2017, 17, 243. 28464813 10.1186/s12906-017-1757-5 PMC5414132

[37] J. Y. Yeo , T. J. Ha , J. S. Nam , M. H. Jung , Biosci. Biotechnol. Biochem. 2011, 75, 2223. 22056454 10.1271/bbb.110538

[38] W. M. Arika , D. W. Nyamai , D. S. Agyirifo , M. P. Ngugi , E. N. M. Njagi , J. Diabetic Complications Med. 2016, 1, https://doi.org/10.4172/2475-3211.1000106.

[39] L. Mahraoui , A. Rodolosse , A. Barbat , E. Dussaulx , A. Zweibaum , M. Rousset , E. Brot‐Laroche , Biochem. J. 1994, 298 *Pt 3*, 629. 8141777 10.1042/bj2980629 PMC1137906

[40] M. L. Chen , N. Sadrieh , L. Yu , AAPS J. 2013, 15, 1043. 23868749 10.1208/s12248-013-9509-z PMC3787221

[41] I. S. Menzies , A. P. Jenkins , E. Heduan , S. D. Catt , M. B. Segal , B. Creamer , Scand. J. Gastroenterol. 1990, 25, 1257. 2125743 10.3109/00365529008998562

[42] C. P. Corpe , M. M. Basaleh , J. Affleck , G. Gould , T. J. Jess , G. L. Kellett , Pflugers Arch. 1996, 432, 192. 8662294 10.1007/s004240050124

[43] L. Rossetti , D. Smith , G. I. Shulman , D. Papachristou , R. A. DeFronzo , J. Clin. Investig. 1987, 79, 1510. 3571496 10.1172/JCI112981 PMC424427

[44] E. Díaz‐de‐Cerio , V. Verardo , A. M. Gómez‐Caravaca , A. Fernández‐Gutiérrez , Int. J. Mol. Sci. 2016, 17, https://doi.org/10.3390/ijms17050699. 10.3390/ijms17050699 PMC4881523 27187352

[45] J. Wruss , P. Lanzerstorfer , S. Huemer , M. Himmelsbach , H. Mangge , O. Hoglinger , D. Weghuber , J. Weghuber , Nutr. J. 2015, 14, 32. 25890155 10.1186/s12937-015-0018-z PMC4396834

[46] S. Alqahtani , L. A. Mohamed , A. Kaddoumi , Exp. Opin. Drug Metab. Technol. 2013, 9, 1241. 10.1517/17425255.2013.802772 23687990

[47] A. M. Nauli , S. M. Nauli , Curr. Clin. Pharmacol. 2013, 8, 247. 23343017 10.2174/1574884711308030012 PMC11957910

[48] D. A. Volpe , Future Med. Chem. 2011, 3, 2063. 22098353 10.4155/fmc.11.149

[49] V. Meunier , M. Bourrie , Y. Berger , G. Fabre , Cell Biol. Toxicol. 1995, 11, 187. 8564649 10.1007/BF00756522

[50] S. Chong , S. A. Dando , R. A. Morrison , Pharma. Res. 1997, 14, 1835. 10.1023/a:1012112820371 9453077

[51] M. Uchida , T. Fukazawa , Y. Yamazaki , H. Hashimoto , Y. Miyamoto , J. Pharmacol. Toxicol. Met. 2009, 59, 39. 10.1016/j.vascn.2008.10.006 19049886

[52] S. Yamashita , K. Konishi , Y. Yamazaki , Y. Taki , T. Sakane , H. Sezaki , Y. Furuyama , J. Pharma. Sci. 2002, 91, 669. 10.1002/jps.10050 11920752

[53] A. Collett , E. Sims , D. Walker , Y. L. He , J. Ayrton , M. Rowland , G. Warhurst , Pharma. Res. 1996, 13, 216. 10.1023/a:1016082829111 8932439

[54] G. Williamson , Mol. Nutr. Food Res. 2013, 57, 48.23180627

[55] R. Cermak , S. Landgraf , S. Wolffram , Br. J. Nutr. 2004, 91, 849. 15182388 10.1079/BJN20041128

[56] K. Johnston , P. Sharp , M. Clifford , L. Morgan , FEBS Lett. 2005, 579, 1653. 15757656 10.1016/j.febslet.2004.12.099

[57] J. B. Park , Biochem. Biophys. Res. Commun. 1999, 260, 568. 10403807 10.1006/bbrc.1999.0890

[58] J. Song , O. Kwon , S. Chen , R. Daruwala , P. Eck , J. B. Park , M. Levine , J. Biol. Chem. 2002, 277, 15252. 11834736 10.1074/jbc.M110496200

[59] A. Barberis , A. Garbetta , A. Cardinali , G. Bazzu , I. D'Antuono , G. Rocchitta , A. Fadda , V. Linsalata , G. D'Hallewin , P. A. Serra , F. Minervini , Biosens. Bioelectron. 2017, 88, 159. 27520503 10.1016/j.bios.2016.08.007

[60] T. C. Chang , S. F. Huang , T. C. Yang , F. N. Chan , H. C. Lin , W. L. Chang , J. Agric. Food Chem. 2007, 55, 1993. 17269785 10.1021/jf062714k

[61] A. L. de la Garza , U. Etxeberria , M. P. Lostao , B. San Roman , J. Barrenetxe , J. A. Martinez , F. I. Milagro , J. Agric. Food Chem. 2013, 61, 12012. 24261475 10.1021/jf4021569

[62] O. El‐Zein , S. I. Kreydiyyeh , Nutrition 2011, 27, 707. 20869203 10.1016/j.nut.2010.07.001

[63] C. Snoussi , R. Ducroc , M. H. Hamdaoui , K. Dhaouadi , H. Abaidi , F. Cluzeaud , C. Nazaret , M. A. Le Gall , J. Nutr. Biochem. 2014, 25, 557. 24656388 10.1016/j.jnutbio.2014.01.006

[64] J. A. Villa‐Rodriguez , E. Aydin , J. S. Gauer , A. Pyner , G. Williamson , A. Kerimi , Mol. Nutr. Food Res. 2017, 61, https://doi.org/10.1002/mnfr.201700566. 10.1002/mnfr.201700566 28868668

[65] E. Diaz‐de‐Cerio , V. Verardo , A. M. Gomez‐Caravaca , Int. J. Mol. Sci. 2016, 17, 699. 10.3390/ijms17050699 PMC4881523 27187352

[66] P. M. Moura , G. H. C. Prado , M. A. A. Meireles , C. G. Pereira , J. Supercrit. Fluids 2012, 62, 116.

[67] H. I. Castro‐Vargas , L. I. Rodriguez‐Varela , S. R. S. Ferreira , F. Parada‐Alfonso , J. Supercrit. Fluids 2010, 51, 319.

