# Supplementary material for: In Vitro and In Vivo Inhibition of Intestinal Glucose Transport by Guava (Psidium Guajava) Extracts
Source: Mol Nutr Food Res. 2018 May 17;62(11):1701012. doi: 10.1002/mnfr.201701012 (PMC6001447; doi:10.1002/mnfr.201701012)

**Supporting information**

**Supplementary Table 1.** Primer sequences for gene expression analysis.

| **Gene** | **Primer forward sequence** | **Primer reverse sequence** |
| --- | --- | --- |
| HPRT1 | 5'-GACCCCACGAAGTGTTGGAT-3' | 5'-ACTGGCGATGTCAATAGGACTC-3' |
| GLUT2 | 5'-AGCTACCGACAGCCTATT-3' | 5'-TGTTTACAGCGCCAACTC-3' |
| GLUT5 | 5'-CGTGCCTGCGATCTTAAT-3' | 5'-GGAGATTCCGAAGACCAAAG-3' |
| SGLT1 | 5'-GCCTCTCAGCCAAGAATATG-3' | 5'-GATAGGCGATGTTGGTACAG-3' |
| CLDN1 | 5'-GCAACCCGTGCCTTGATG-3 | 5'-GCCAGTGAAGAGAGCCTGAC-3' |
| CLDN3 | 5'-CCAACACCATTATCCGGGAC-3' | 5'-GTAGTCCTTGCGGTCGTAG-3' |
| OCLN | 5'-TGTGGGACAAGGAACACAT-3' | 5'-TGCCATTGGAAGAGTATGC-3' |

**Supplementary Table 2.** Mean C_t_ values for genes under study obtained with qPCR analysis.

| **Mean C_t_** | | | | | | |
| --- | --- | --- | --- | --- | --- | --- |
| *Target* | *Day 2* | *Day 3* | *Day 4* | *Day 5* | *Day 6* | *Day 7* |
| **HPRT1** | 24,79 | 25,06 | 24,65 | 25,58 | 25,46 | 25,02 |
| **GLUT2** | 26,24 | 27,27 | 27,52 | 28,45 | 27,71 | 27,12 |
| **GLUT5** | 33,16 | 32,76 | 29,37 | 28,25 | 26,99 | 25,43 |
| **SGLT1** | 28,57 | 29,47 | 27,20 | 27,41 | 26,23 | 26,10 |
| **CLDN1** | 26,21 | 25,28 | 24,22 | 24,91 | 24,56 | 24,12 |
| **CLDN3** | 25,78 | 24,15 | 23,08 | 23,87 | 23,51 | 23,23 |
| **OCLN** | 21,56 | 22,13 | 21,13 | 22,40 | 22,31 | 21,50 |

**Supplementary Figure 1.** Effects of the intestinal glucose transport inhibitors phloretin and phloridzin in fast differentiated Caco-2 cells. Cells were grown (for 5 days) on collagen coated 0.4 µm transwell inserts for monolayer formation and fast differentiation. On day 5-7, the inhibitory effect of the indicated substances on the intestinal glucose transport across the cell monolayer was tested. Cell culture medium with 2.1 g/L glucose and 1 g/L xylitol as well as the respective substances were used as donor solution in the apical compartment. Samples were taken from the basolateral compartment (HEPES buffer) at the respective time points. Glucose concentration of the samples was measured by HPLC. (A) Effect of 100 mg/L phloretin and phloridzin on intestinal glucose transport through the differentiated Caco-2 monolayer. (B) Respective calculated P_app_ values.

**
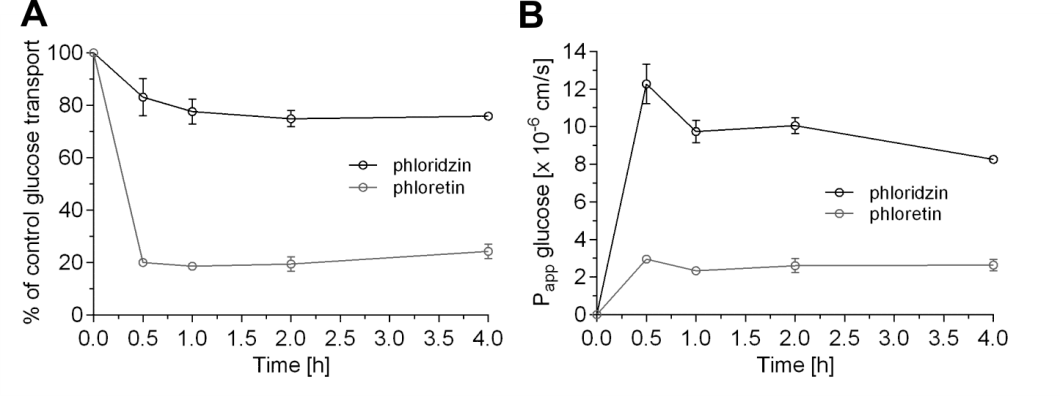
**

**Supplementary Figure 2.** Gene expression analysis of relevant sugar transporters (GLUT2, GLUT5 and SGLT1) and tight junction proteins (claudin-1, CLDN1; claudin-3, CLDN3; occludin, OCLN). Total RNA was isolated from Caco-2 cells in triplicates on differentiation days 2-7. After reverse transcription to cDNA, qPCR was executed. (A-F) Data was normalized to the housekeeping gene HPRT1 and calculated to relative mRNA expression levels using the 2-∆∆cT method. Error bars are based on the standard deviation (n = 3). qPCR-products were analyzed via agarose-gel electrophoresis resulting in explicit bands.

**
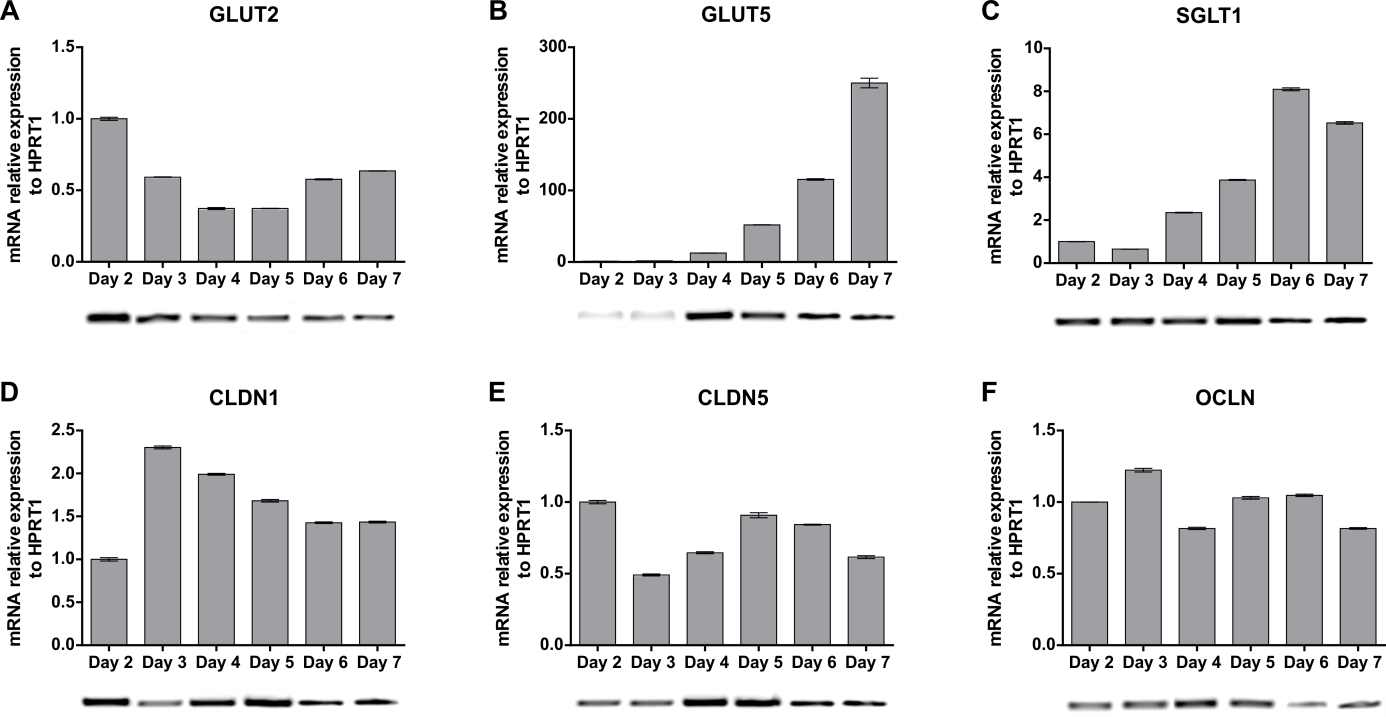
**

**Supplementary Figure 3.** Effect of the 12 hours fasting period on body weight and blood glucose levels of mice under study. Body weight (A) as well as the blood glucose level (B) was determined before and after a 12 hours period of food withdrawal to ensure comparable physiological parameters.


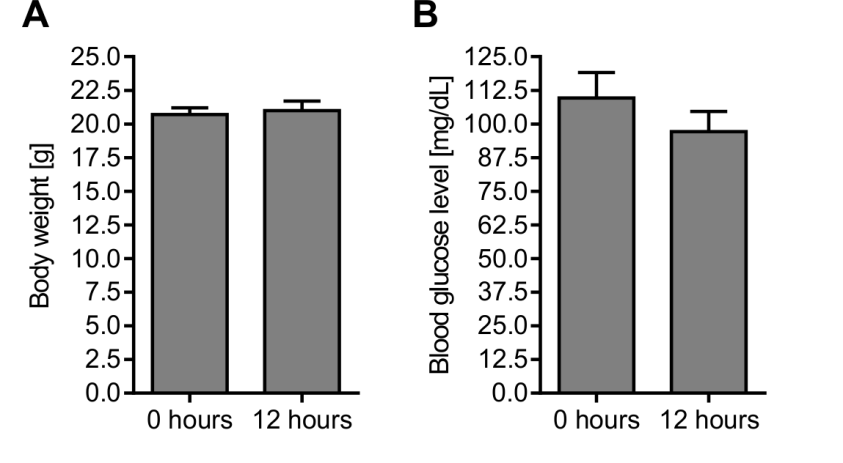

Supplement: Supplementary file 1 — Supporting Information [file MNFR-62-na-s001.docx]
