# Supplementary figures and images for: In Vitro and In Vivo Inhibition of Intestinal Glucose Transport by Guava (Psidium Guajava) Extracts
Source: Mol Nutr Food Res. 2018 May 17;62(11):1701012. doi: 10.1002/mnfr.201701012 (PMC6001447; doi:10.1002/mnfr.201701012)

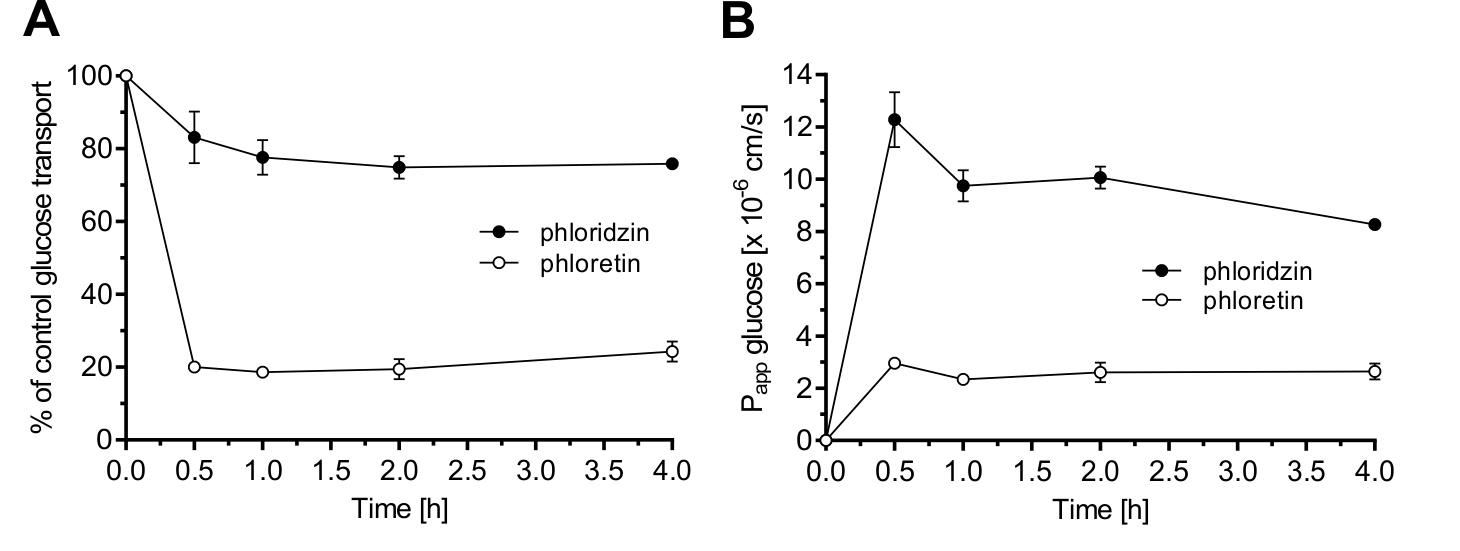

Supplement: Supplementary file 2 — Supporting Information Figure S1 [file MNFR-62-na-s002.tif]

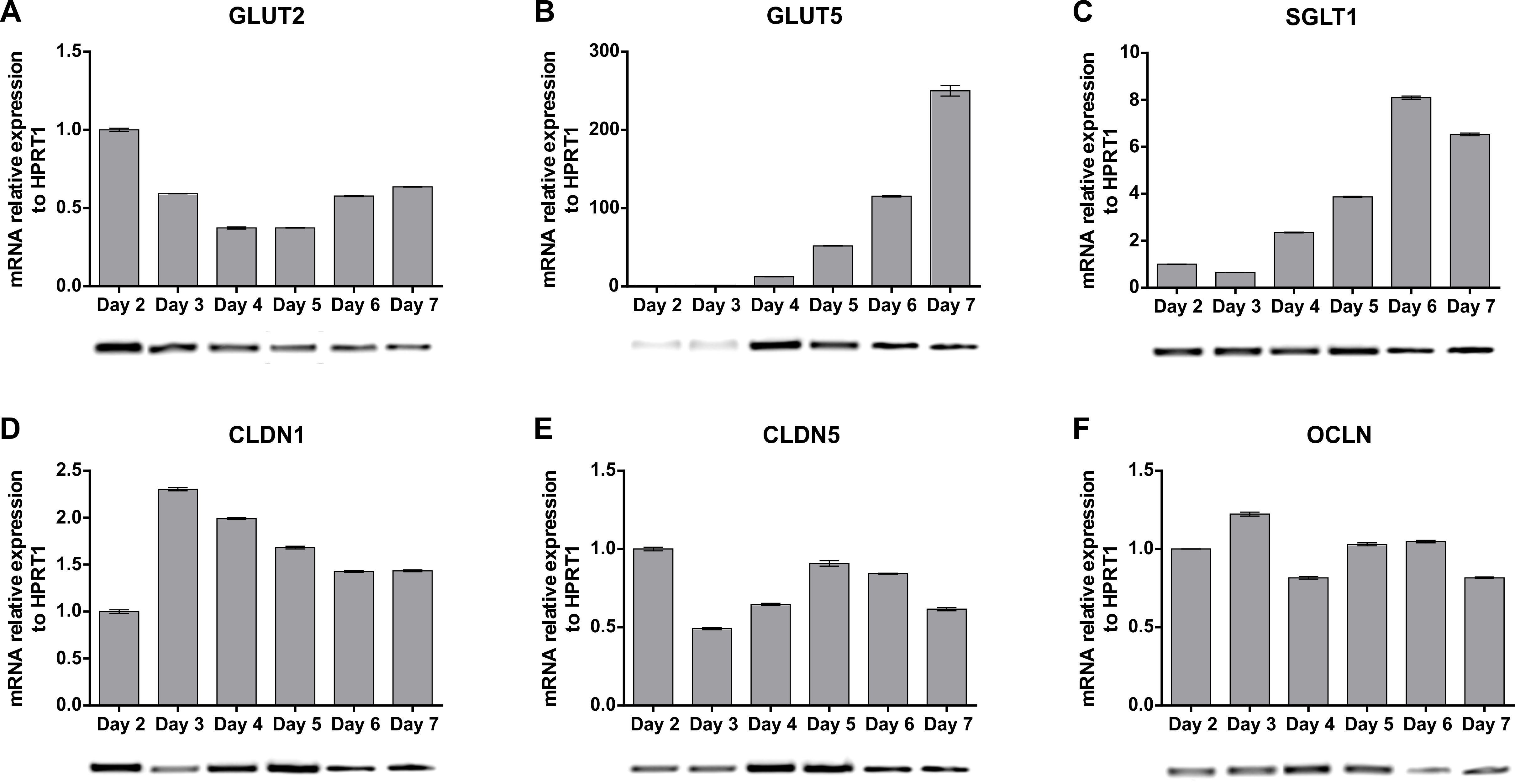

Supplement: Supplementary file 3 — Supporting Information Figure S2 [file MNFR-62-na-s003.tif]

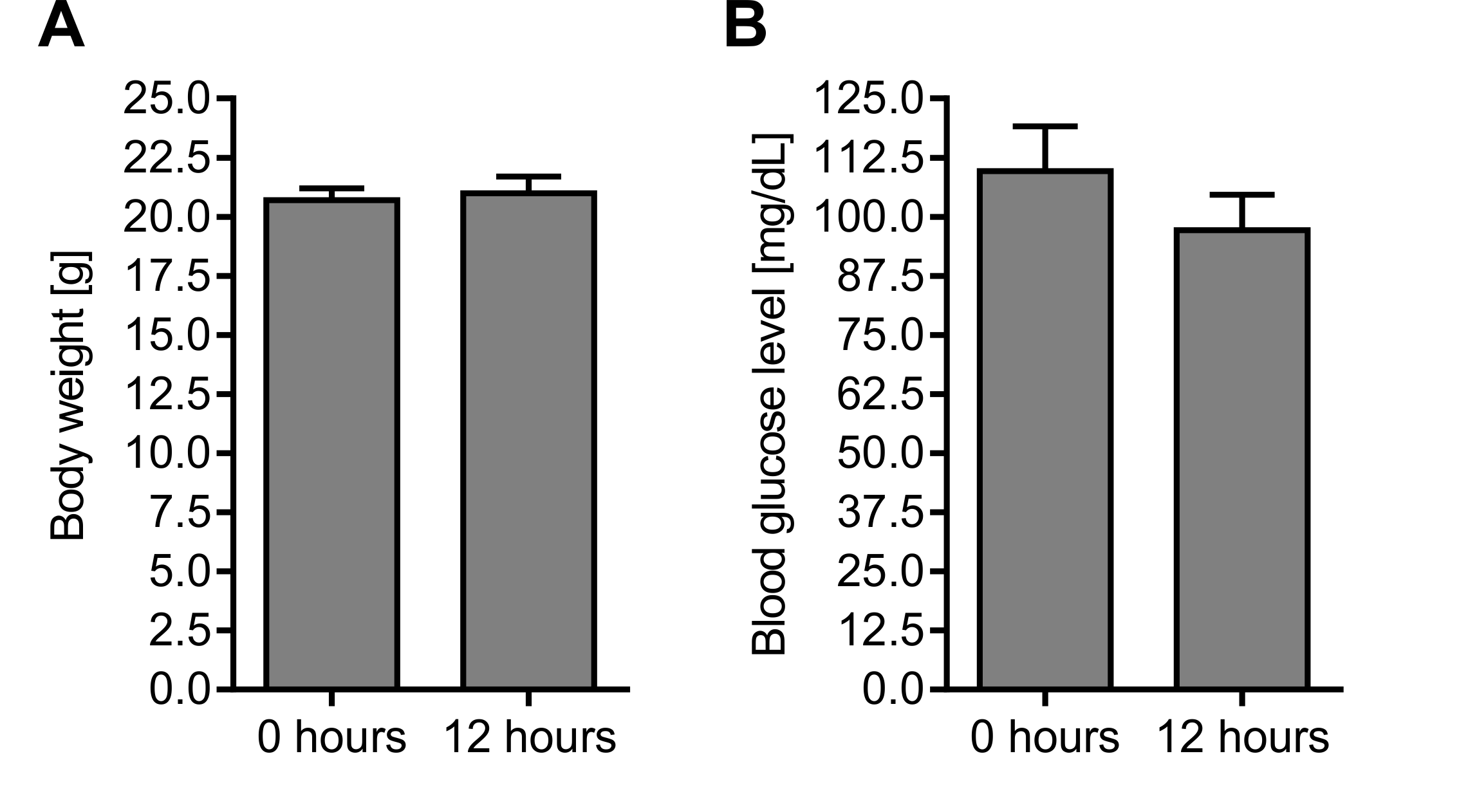

Supplement: Supplementary file 4 — Supporting Information Figure S3 [file MNFR-62-na-s004.tif]
